# USMLE Scores Predict Success in ABEM Initial Certification: A Multicenter Study

**DOI:** 10.5811/westjem.2016.12.32478

**Published:** 2017-02-07

**Authors:** Elie Harmouche, Nikhil Goyal, Ashley Pinawin, Jumana Nagarwala, Rahul Bhat

**Affiliations:** * Henry Ford Hospital, Department of Emergency Medicine, Detroit, Michigan; † Henry Ford Hospital, Department of Emergency Medicine and Internal Medicine, Detroit, Michigan; ‡ MedStar Georgetown University Hospital/MedStar Washington Hospital Center, Department of Emergency Medicine, Washington, DC

## Abstract

**Introduction:**

There are no existing data on whether performance on the United States Medical Licensing Examination (USMLE) predicts success in American Board of Emergency Medicine (ABEM) certification. The aim of this study was to determine the presence of any association between USMLE scores and first-time success on the ABEM qualifying and oral certification examinations.

**Methods:**

We retrospectively collected USMLE Step 1, Step 2 Clinical Knowledge (CK) scores and pass/fail results from the first attempt at ABEM qualifying and oral examinations from residents graduating between 2009 and 2011 from nine EM programs. A composite score was defined as the sum of USMLE Step 1 and Step 2 CK scores.

**Results:**

Sample was composed of 197 residents. Median Step 1, Step 2 CK and composite scores were 218 ([IQR] 207–232), 228 (IQR 217–239) and 444 (IQR 427–468). First-time pass rates were 95% for the qualifying examination and 93% for both parts of the examination. Step 2 CK and composite scores were better predictors of achieving ABEM initial certification compared to Step 1 score (area under the curve 0.800, 0.759 and 0.656). Step 1 score of 227, Step 2 CK score of 225 and composite score of 444 predicted a 95% chance of passing both boards.

**Conclusion:**

Higher USMLE Step 1, Step 2 CK and composite scores are associated with better performance on ABEM examinations, with Step 2 CK being the strongest predictor. Cutoff scores for USMLE Step 1, Step 2 CK and composite score were established to predict first-time success on ABEM initial certification.

## INTRODUCTION

Residencies in emergency medicine (EM) teach the fundamental skills, knowledge, and humanistic qualities that constitute the foundations of EM practice.^1^ Certification by the American Board of Emergency Medicine (ABEM) is an important step in ensuring that graduates of EM residency programs are able to practice independently and to ensure the highest standards in the specialty.^2^ Recognizing this, the Accreditation Council for Graduate Medical Education (ACGME) requires that 80% of graduates of an EM residency program from the preceding five years must pass the ABEM certification examination on their first attempt.^1^ Passing the ABEM certification examinations is also a Level 5 educational milestone for all EM residents.^3^ Therefore, obtaining successful ABEM certification on the first attempt is an important goal for EM program directors, residents, oversight agencies, and the public.

EM program directors invest a significant amount of time and effort each year to select medical students who will become successful, ABEM-certified EM practitioners. In 2014, 4,101 medical students applied to 171 EM residency programs offering 1,821 positions, with each program receiving an average of 807 applications.^4^,^5^ With increasing competition, program directors use a number of metrics to identify the best candidates for matriculation into their residency program. Scores on the United States Medical Licensing Examination (USMLE) are commonly used by EM program directors to select applicants.^6^ The USMLE is a three-step examination that assesses a physician’s ability to apply knowledge, concepts, and principles and to demonstrate fundamental patient-centered skills that are important in health and disease and that constitute the basis of safe and effective patient care.^6^,^7^ The USMLE examinations are widely considered valid measures of a medical student’s medical knowledge.^8^

Certain specialties have studied the correlation between USMLE scores and board certification rates. Diagnostic radiology, obstetrics and gynecology, pathology, pediatrics, physical medicine and rehabilitation, and surgery have all shown that high USMLE Step 1 or Step 2 Clinical Knowledge (CK) scores may predict successful board certification.^9^–^14^ Variable data have been reported by orthopedic surgery and internal medicine.^15^–^21^ A search of the literature showed no reports examining for correlation between USMLE scores and ABEM certification rates for EM, despite the widespread use of USMLE scores in medical knowledge assessment and resident selection.^6^,^22^ The aim of this study was to determine if USMLE Step 1 or Step 2 CK scores are associated with successful first-attempt ABEM initial certification.

## METHODS

### Study Design

This retrospective cohort study examined data from residents who graduated from nine ACGME-accredited EM residency programs between 2009 and 2011. Data were originally collected via a multi-institutional collaborative effort, details of which have been described previously.^23^ Briefly, nine programs volunteered to provide performance data on all their residents graduating in the previous three years, along with pre-residency predictor data available at the time of resident matriculation into the program. Institutional review board approval was obtained at each participating program as required.

Residents’ three-digit USMLE Step 1 and Step 2 CK scores were obtained from their residency files. If a resident had multiple attempts at any examination, the first score was used. A “composite USMLE score” was defined as the sum of the USMLE Step 1 and Step 2 CK score. Pass or fail status of the resident’s first attempt on the ABEM qualifying examination (“written boards”) and the ABEM oral certification examination (“oral boards”) were collected and used as outcome variables. We excluded residents with no USMLE scores reported (e.g., osteopathic students), those who had not taken their written or oral boards, and those whose scores were not available at the time of data collection.

**Population Health Research Capsule**

What do we already know about this issue?

The USMLE examinations are widely considered valid measures of a medical student’s medical knowledge and may possibly correlate with ABEM certification.

What was the research question?

The aim of this study was to determine if there is any association between USMLE scores and first-time success on the ABEM Qualifying and Oral Certification Examinations.

What was the major finding of the study?

Higher USMLE Step 1, Step 2 CK and composite scores are associated with better performance on ABEM examinations with Step 2 CK being the strongest predictor.

### Data Analysis

Wilcoxon rank sum test evaluated the association between USMLE scores and written and oral boards outcomes. P-values < .05 were considered significant. We used univariate logistic regression analysis to obtain receiver operating characteristic area under the curve when using the USMLE scores to predict boards outcomes. USMLE score cutoff points based on 90%, 95%, and 99% chance of passing the boards were defined as the optimal cutoff points for predicting boards outcomes. We performed data analysis using SAS 9.4 (SAS Institute, Cary, NC, USA).

## RESULTS

The nine EM programs were mostly urban, academic centers with eight to 14 residents in each class and emergency department censuses ranging from 60,000 to 115,000 patients per year.^23^ Data were available for 286 residents; eight did not have a USMLE Step 1 score, 60 did not have a USMLE Step 2 score, 21 did not have a written board status available, and 26 did not have oral board results. We included a total of 197 residents with complete data in the analysis. Of these, 187 (95%) residents passed the written boards on their first attempt, 193 (98%) passed the oral boards, and 183 (93%) passed both boards on their first attempt. USMLE Step 1, Step 2, and composite scores in addition to first-attempt board pass rate are listed in Table 1. Residents who passed written boards had significantly higher USMLE Step 1, Step 2, and composite USMLE scores (p < .001), while residents who passed both boards had significantly higher Step 2 and composite scores (p < .001) (Table 2).

We calculated receiver operating characteristic area under the curve value for each score and determined predictor cutoff points. Table 3 shows the association of Step 1, Step 2, and composite scores compared to passing written and both parts of the ABEM examination.

## DISCUSSION

Our study of EM residents across nine centers showed that those who had better performance on the USMLE Step 1 and Step 2 CK examinations had a higher probability of obtaining ABEM initial certification at the first attempt. We found that a USMLE Step 1 score of 202 and Step 2 CK score of 209 or higher correlated with a 90% chance of passing the ABEM qualifying examination on the first attempt, and scores of 205 and 215, respectively, correlated with a 90% chance of achieving first-attempt ABEM initial certification.

Prior studies in EM have attempted to identify pre-residency variables that may predict success during residency. Election into the Alpha Omega Alpha honor society, grade assigned for EM rotation, USMLE Step 1 score, interview score, letters of recommendation, scholarly activity, quality of medical school, and distinctive talents have all demonstrated association with successful performance during residency or high placement on rank lists.^23^–^26^ Many of these factors are used by EM program directors while selecting and ranking applicants.^6^ To identify USMLE scores that would identify candidates with a high likelihood of passing the ABEM examinations on first attempt, the 2014 pass rates of 90% on first-attempt written boards and 96% on first-attempt oral boards prompted our use of the 90%, 95%, and 99% cutoff points for the Step 1, Step 2, and composite scores, respectively.^27^ While studies correlating USMLE and ABEM are lacking, the cutoff scores identified by our study appear to correlate well with previous reported scores among different specialties. USMLE scores below 200 correlated with higher chance of failure in the American Board of Obstetrics and Gynecology, while scores less than 204 and 207 correlated with higher chance of failure in orthopedic and general surgery board examinations.^13^,^16^,^20^ In addition, we found that the Step 2 CK score was most useful in predicting which students had a higher likelihood of passing their board examinations. Again, these findings seem to be reproducible among different specialties, notably general surgery, orthopedics, and obstetrics and gynecology, where Step 2 scores were superior to Step 1 scores in determining board outcomes.^20^,^28^,^29^

These results suggest that USMLE scores can play an important role in screening applicants to interview for EM programs. In addition, these results may help identify EM residents at risk for not achieving first-attempt ABEM initial certification and may be used to structure individualized learning plans during residency.

## LIMITATIONS

Limitations of our study include the retrospective design leading to reporting bias and incomplete records. The study sample may not be representative of the entire population because of a lack of a systematic enrollment process. (All programs that volunteered to participate were enrolled.) Data for 89 residents were incomplete or missing, and some programs did not record their residents’ USMLE Step 2 CK scores after the match. We did assess excluded residents who showed a higher mean Step 1 score compared to study residents (225 vs. 219; p = .004), and no statistically significant difference was detected in mean Step 2 and composite scores between excluded and included residents (225 vs. 228 and 445 vs. 447; p = .4 and .3, respectively). The first-time pass rate for ABEM qualifying, oral, and both examinations for excluded residents was 95%, 98%, and 95%, respectively, compared to 95%, 98%, and 93% in included residents, respectively. Based on these observations, excluded residents seemed to follow similar trends compared to included residents; however, we do not expect that excluding these residents would affect internal validity of our presented data. There was only one resident with multiple attempts on any USMLE examinations; this resident had two attempts on USMLE Step 1 with scores of 178 (Fail) and 194 (Pass), and only the first score was included per study protocol.

ABEM calculates a numerical score for performance on their examinations, but these data are shared only with individual residents and not EM program directors. Therefore, our study treated performance on the ABEM examinations as a categorical variable (pass/fail) as opposed to a continuous one.

An additional limitation of our study is that the number of residents failing the board examination was low and our cohort had a higher pass rate in both boards compared to 2014 nationwide rates for ABEM.^27^ Hence, there might be a selection bias as residents included may not accurately represent the entire population of EM residents nationwide. ABEM pass/fail data are only available for residents who successfully complete an EM residency program. Therefore, it is impossible to conduct this study in a more relevant sample, such as all applicants to an EM program.

All included residents took their USMLE Step 1 between 2004 and 2006 and Step 2 between 2006 and 2008. USMLE scores for a given step are comparable across years and across forms; however, the USMLE cautions against comparing scores that were obtained at dramatically different periods of time.^30^ Therefore, it may be difficult to apply our calculated cutoff scores to medical students seeking EM residencies today. Mean scores on USMLE examinations taken by 2014 graduates from U.S./Canadian medical schools are slightly higher than those from preceding years. While this is the first study to include multiple ACGME-accredited EM residency programs, future studies should focus on a larger number of community and academic programs and aim to include more residents. Future areas of research should also focus on patient-centered outcomes, as there are limited data on how performance on USMLE or ABEM examinations relates to clinical outcomes.

## CONCLUSION

In our multicenter study, higher USMLE Step 1, Step 2 CK, and composite scores were associated with better performance on ABEM examinations with Step 2 CK being the strongest predictor. Cutoff scores for USMLE Step 1, Step 2 CK, and composite score were established to predict first-time success on ABEM initial certification.

## Figures and Tables

**Table 1:** USMLE scores for emergency medicine residents included in a study of the correlation between scores and initial ABEM certification.

	USMLE step 1	USMLE step 2 CK	Composite score
Mean ± SD	219 ± 16	228 ± 17	447 ± 30
Median [IQR1–IQR3]	218 [207–232]	228 [217–239]	444 [427–468]

*ABEM*, American Board of Emergency Medicine; *CK*, clinical knowledge; *IQR*, interquartile range; *SD*, standard deviation; *USMLE,* United States Medical Licensing Examination.

**Table 2:** Association of USMLE step score with ABEM boards outcome.

	ABEM qualifying examination			ABEM qualifying and oral examination		
				
	Fail (n=10)	Pass (n=187)	p-value	Fail (n=14)	Pass (n=183)	p-value
USMLE step 1	206 ± 8	220 ± 16	<.001*	212 ± 16	220 ± 16	.072
USMLE step 2 CK	203 ± 15	229 ± 16	<.001*	210 ± 18	229 ± 16	<.001*
Composite USMLE score	410 ± 20	449 ± 29	<.001*	421 ± 30	449 ± 29	<.001*

*ABEM*, American Board of Emergency Medicine; *CK*, clinical knowledge; *SD*, standard deviation; *USMLE,* United States Medical Licensing Examination.

All scores are represented as mean ± standard deviation.

* Statistically significant, p < .05.

**Table 3:** Variables predicting first-attempt success at ABEM certification.

	ABEM qualifying examination				ABEM qualifying and oral examination			
		
		Cutoff scores predicting success				Cutoff scores predicting success		
				
	AUC (95% CI)	90% PPV	95% PPV	99% PPV	AUC (95% CI)	90% PPV	95% PPV	99% PPV
USMLE step 1	0.770 (0.668, 0.872)	202	213	239	0.656 (0.494, 0.818)	205	227	not attained
USMLE step 2 CK	0.883 (0.772, 0.994)	209	216	232	0.800 (0.664, 0.935)	215	225	247
Composite score	0.878 (0.777, 0.979)	417	428	451	0.759 (0.604, 0.913)	426	444	485

*ABEM*, American Board of Emergency Medicine; *AUC,* area under the receiver operating characteristic curve; *CK*, clinical knowledge; *PPV,* positive predictive value; *USMLE,* United States Medical Licensing Examination.
